# Persistent Innate Immune Stimulation Results in IRF3-Mediated but Caspase-Independent Cytostasis

**DOI:** 10.3390/v12060635

**Published:** 2020-06-11

**Authors:** Christian Urban, Hendrik Welsch, Katharina Heine, Sandra Wüst, Darya A. Haas, Christopher Dächert, Aparna Pandey, Andreas Pichlmair, Marco Binder

**Affiliations:** 1 Institute of Virology, School of Medicine, Technical University of Munich, 81675 Munich, Germany; c.urban@tum.de (C.U.); darya.haas@tum.de (D.A.H.); 2 Research Group “Dynamics of Early Viral Infection and the Innate Antiviral Response”, Division Virus-Associated Carcinogenesis (F170), German Cancer Research Center (DKFZ), 69120 Heidelberg, Germany; h.welsch@dkfz-heidelberg.de (H.W.); k.heine@stud.uni-heidelberg.de (K.H.); s.wuest@dkfz-heidelberg.de (S.W.); c.daechert@dkfz-heidelberg.de (C.D.); Aparna.Pandey@fmi.ch (A.P.); 3 Faculty of Biosciences, Heidelberg University, 69117 Heidelberg, Germany; 4 German Center for Infection Research (DZIF), Munich Partner Site, 81675 Munich, Germany

**Keywords:** innate immunity, RIG-I, MAVS, IRF3, interferon, HCV, cytostasis

## Abstract

Persistent virus infection continuously produces non-self nucleic acids that activate cell-intrinsic immune responses. However, the antiviral defense evolved as a transient, acute phase response and the effects of persistently ongoing stimulation onto cellular homeostasis are not well understood. To study the consequences of long-term innate immune activation, we expressed the NS5B polymerase of Hepatitis C virus (HCV), which in absence of viral genomes continuously produces immune-stimulatory RNAs. Surprisingly, within 3 weeks, NS5B expression declined and the innate immune response ceased. Proteomics and functional analyses indicated a reduced proliferation of those cells most strongly stimulated, which was independent of interferon signaling but required mitochondrial antiviral signaling protein (MAVS) and interferon regulatory factor 3 (IRF3). Depletion of MAVS or IRF3, or overexpression of the MAVS-inactivating HCV NS3/4A protease not only blocked interferon responses but also restored cell growth in NS5B expressing cells. However, pan-caspase inhibition could not rescue the NS5B-induced cytostasis. Our results underline an active counter selection of cells with prolonged innate immune activation, which likely constitutes a cellular strategy to prevent persistent virus infections.

## 1. Introduction

The innate immune system is highly efficient in preventing and clearing the majority of virus infections and therefore constitutes an essential barrier inhibiting viral spread within populations and across species [1]. Most viruses developed sophisticated mechanisms to escape cellular defense systems [2]. Masters of such adaptations are viruses that manage to persist in their host despite presence of a fully functional innate immune system. How such viruses escape surveillance by the innate immune system is only partially understood, particularly for RNA viruses, such as the Hepatitis C virus (HCV), since these viruses continuously generate viral nucleic acids that activate innate immune pattern recognition receptors (PRRs). One PRR capable of selectively sensing non-self RNA species, such as uncapped 5′-triphosphate-dsRNA generated during viral genome replication, is the RNA helicase retinoic acid inducible gene I (RIG-I) [3]. Upon dsRNA binding, RIG-I induces aggregation of mitochondrial antiviral signaling protein (MAVS) on the outer mitochondrial membrane [4]. MAVS aggregation in turn triggers further downstream signaling by inducing activation and nuclear translocation of the transcription factors interferon regulatory factor 3 (IRF3) and nuclear factor kappa B (NFκB) [5]. PRR engagement subsequently leads to the production of type-I (α/β) and -III (λ) interferons (IFNs), which upon auto and paracrine signaling induce the expression of a large panel of IFN-stimulated genes (ISGs) with antiviral function. Expression of type-I interferons and downstream signaling are very tightly regulated at various levels. First, IFNs are only induced after engagement of nucleic acid signatures that are characteristic of viral infections [2]. Second, negative feedback loops are in place that shut off innate immune signaling and return the cell to a homeostatic state [6]. It is unclear, however, how the persistent presence of viral pathogen-associated molecular patterns (PAMPs) influence activation of the innate immune response. This point is difficult to address experimentally since most viruses acquired mechanisms that target critical signaling pathways. The HCV NS3/4A protease, for instance, prevents activation of interferon induction by cleaving MAVS, thereby impairing proper signal transduction downstream of RIG-I [7,8]. Nevertheless, some persistent viruses, including HCV itself, still induce high amounts of antiviral cytokines—indicating successful detection of the viral pathogen and induction of antiviral immunity [9,10,11]. Here we asked whether the long-term presence of immune-stimulatory nucleic acids, as it is commonly seen in persistent virus infections, may reshape the innate immune system to induce a tolerogenic state in which the innate immune system is paralyzed. Such a system would on the one hand be essential for the host to prevent exaggerative tissue damage, but could as well be beneficial for the virus, ultimately preventing its clearance.

## 2. Materials and Methods

### 2.1. PCR and Gateway Cloning

NS5B-eGFP recombinant DNA was generated by amplifying sequences for NS5B and eGFP using PCR and subsequently fusing both fragments in an overlap extension PCR approach (Q5 High-Fidelity PCR Kit, NEB, Ipswich, MA, USA). The fused sequence harbored attB sites at both ends to permit Gateway cloning into entry vector pDONR207 and subsequently into destination vector pWPI_BLR_rfB following the manufacturer protocol (Gateway BP and LR Clonase II, Life Technologies, Carlsbad, CA, USA). Plasmids were transformed into and amplified in chemo-competent *Escherichia coli* DH5α (Heidelberg University, Heidelberg, Germany) cultured at 37 °C in LB medium (1% Bacto-Trypton, 0.5% Yeast extract, 0.5% NaCl) supplemented with the respective selection antibiotic (100 µg/mL Carbenicillin, Sigma Aldrich, Munich, Germany; 7 µg/mL Gentamicin, Life Technologies).

### 2.2. Cell Culture

Cells were cultured at 37 °C and 95% humidity in the presence of 5% CO_2_ in Dulbecco’s modified eagle medium (DMEM high glucose, Life Technologies) supplemented with a final concentration of 10% fetal calf serum (FCS, Thermo Fisher Scientific, Waltham, MA, USA), 1× non-essential amino acids (Thermo Fisher Scientific) as well as 100 U/mL penicillin and 100 ng/mL streptomycin (Life Technologies). Cells were passaged at 80% confluence in a 1:10 ratio. For detachment, 0.05% Trypsin-EDTA (Life Technologies) was used.

### 2.3. Cell Line Generation, Lentivirus Production, Transduction, and Transfection

Transgene expressing cell lines were generated by lentiviral transduction. Lentiviral particles were produced by transfecting HEK293T cells (DKFZ, Heidelberg, Germany) with plasmids pCMV-dr8.91, pMD2.G and the respective retroviral vector (pWPI) in a 3:1:3 ratio using calcium phosphate transfection (CalPhos Mammalian Transfection Kit, Takara Bio Europe, Saint-Germain-en-Laye, France). Supernatant of particle producing cells was harvested and sterile filtered 2 days after transfection. A549 cells (Heidelberg University Hospital, Heidelberg, Germany) were treated two times for 12 h with particle-bearing supernatant containing 10 µg/mL polybrene (Merck Millipore, Darmstadt, Germany). Afterwards, medium was changed to complete DMEM supplemented with the appropriate selection antibiotic to select for transgene expressing cells (5 µg/mL blasticidin, MP Biomedicals, Santa Ana, CA, USA; 1 µg/mL puromycin, Sigma Aldrich; or 1 mg/mL geneticin (G418), Santa Cruz, Dallas, TX, USA). The A549 IRF3 knock-out cell line was generated by CRISPR/Cas9 technology. In brief, DNA oligonucleotides coding for a guide RNA against exon 3 of human IRF3 (sense: 5′-CACCCGGAAATTCCTCTTCCAGGT-3′; antisense: 5′-AAACACCTGGAAGAGGAATTTCCG-3′) were cloned into expression vector LentiCRISPRv2 (Feng Zhang, Addgene #52961) following the associated protocol (lentiCRISPRv2 and lentiGuide oligo cloning protocol) to generate LentiCrisprV2_Puro_IRF3. A549 wild-type (WT) cells were transduced with LentiCrisprV2_Puro_IRF3 and selected with puromycin. IRF3 knock-out efficiency in the cell pool was validated by Western blot with anti-IRF3 antibody. Next, cells were seeded in limiting dilution (0.5 cells/well) on 96-well plates and cultured under selection. Single cell clones were again validated by Western blot and Sanger sequencing for complete IRF3 knock-out. In this study, IRF3^−/−^ clone 1.1 was used and A549 cells transduced with a lentiCRISPRv2 plasmid coding for a non-targeting guide RNA served as control in experiments with IRF3 and MAVS knock-out. Generation of HEK-FlpIn-SH-MAVS and -GFP cells was performed as described earlier [12] and transgene expression was induced by treatment with 1 µg/mL doxycycline (Sigma-Aldrich). Transfection of 1 µg poly-I:C (Sigma-Aldrich) into 1 × 10^6^ HEK-FlpIn-SH-MAVS or -GFP cells was performed using Lipofectamine 2000 (Invitrogen, Carlsbad, CA, USA) according to the manufacturer’s protocol. A549 RIG-I and A549 MAVS knock-out cells have been generated previously [13,14], A549-IFIT1-eGFP cells [15] were a kind gift of Prof. Dr. Ralf Bartenschlager (Heidelberg University), and PH5CH8 cells were kindly provided by Dr. Volker Lohmann (Heidelberg University).

### 2.4. RNA Extraction and qRT-PCR

RNA isolation (NucleoSpin® RNA Plus, Macherey-Nagel, Düren, Germany), cDNA synthesis (High-Capacity cDNA Reverse Transcription Kit, Applied Biosystems, Waltham, MA, USA), and quantitative PCR (qPCR; iTaq™ Universal SYBR® Green Supermix, Bio-Rad, Hercules, CA, USA) were performed according to manufacturer’s protocols. Fold changes of target genes were calculated relative to GAPDH using the 2^−ΔΔCt^ or 2^−ΔCt^ method [16].

### 2.5. Protein Extraction, SDS-PAGE, and Western Blot

Cells were washed in PBS, lysed in 1× Laemmli buffer (16.7 mM TRIS pH 6.8, 5% glycerol, 0.5% SDS, 1.25% β-mercaptoethanol, 0.01% bromophenol blue) at 95 °C for 5 min and cleared from debris. Then, 5 × 10^4^ cells were loaded onto an SDS-polyacrylamide gel (8% acrylamide:bisacrylamide (29:1), 0.1% TEMED, 0.1% saturated ammonium persulfate solution, 0.375 M Tris Base pH 8.8, 0.1% SDS), and run at 120 V for 60–90 min in 1× TGS (25 mM Tris Base pH 8.3, 192 mM glycine, 0.1% SDS) to separate proteins. Transfer of proteins onto a methanol-activated PVDF membrane (Bio-Rad) was performed with the Bio-Rad Semi Dry blotting system and 1× Semi Dry transfer buffer (25 mM Tris Base pH 8.3, 150 mM glycine, 10% methanol) at 25 V and 1 A for 30 min. Membranes were dried, rehydrated in ethanol, washed in PBS-T (PBS with 0.1% Tween-20), and blocked in PBS-T supplemented with 5% milk (Roth) for at least 30 min. Incubation with primary antibodies at 4 °C overnight was followed by four 15 min washing steps with PBS-T. Incubation with secondary antibodies at room temperature for 1 h was followed by four 15 min washing steps with PBS-T. Protein bands were visualized in an ECL Chemocam (Intas Science Imaging Instruments GmbH, Göttingen, Germany) or Bio-Rad ChemiDox XRS+ Imager (Bio-Rad) using Clarity Western ECL Substrate (Bio-Rad) or Western Lightning Plus-ECL (PerkinElmer, Waltham, MA, USA). Quantification of protein bands was performed with ImageLab (Bio-Rad).

### 2.6. Flow Cytometry and Fluorescence-Activated Cell Sorting

Cells were washed in FACS buffer (1× PBS supplemented with 1% FCS) and fixated in Cytofix/Cytoperm solution (BD Biosciences, Franklin Lakes, NJ, USA) for 20 min at 4 °C in the dark. Afterwards, cells were washed twice with 1× Perm/Wash buffer (BD Biosciences). Intracellular staining of IFIT1 was performed by incubating cells with anti-IFIT1 (1:500 in 1× Perm/Wash) for 30 min on ice. After two washing steps with Perm/Wash, cells were incubated with a fluorophore-coupled secondary antibody (anti-rabbit Alexa Fluor 647, 1:1000 in 1× Perm/Wash) for 30 min on ice and protected from light. Finally, cells were again washed twice with 1× Perm/Wash and resuspended in FACS buffer. Flow cytometry was performed using a LSRFortessa flow cytometer equipped with the FACSDiva software (BD Biosciences). Data analysis was performed in FlowJo 10 and R. For FACS sorting, cells were washed once in PBS, resuspended in FACS buffer, and sorted into several fractions based on their NS5B-eGFP levels. After sorting, cells were seeded in complete DMEM. 

### 2.7. Sendai Infection Assay

A549 cells were infected 10 h after seeding with Sendai virus (kind gift from Rainer Zawatzky, DKFZ, Heidelberg) at an MOI of 0.0038 and harvested 16 h post infection for RNA extraction.

### 2.8. Cell Growth Assay

Cells were transduced as described above to express NS5B-eGFP alone or together with HCV NS3/4A and seeded at 2 × 10^3^ cells per 96-well 5 days post transduction. Pan-caspase inhibitor Z-VAD-FMK (R&D Systems, Minneapolis, MN, USA) and necroptosis inhibitor Necrostatin 7 (Sigma Aldrich) were added at the time of seeding. A549-IFIT1-eGFP cells were seeded at 4 × 10^3^ cells per 96-well and treated with IFN-α (recombinant human IFN-α B/D, kind gift of Prof. Dr. Peter Stäheli), IFN-β (IFN-β1, Bioferon GmbH & Co, Laupheim, Germany), IFN-λ (IFN-λ1, PeproTech, Hamburg, Germany) or the supernatant of HEK-FlpIn-MAVS cells at 6 h post seeding. For supernatant transfer, MAVS expression in HEK-FlpIn-MAVS cells was induced for 1 day with 1 µg/mL doxycycline, medium was harvested and cleared from cells at 800 × g, 1:4 diluted with complete DMEM and added to A549-IFIT1-eGFP cells. Cell growth of HEK-FlpIn-MAVS cells upon HCV NS3/4A expression was monitored following NS3/4A transduction and selection for 5 days. For this purpose, HEK-FlpIn-MAVS-NS3/4A cells were seeded at 6 × 10^3^ cells per 96-well and treated with 1 µg/mL doxycycline. For each experiment, cell growth as well as IFIT1-eGFP expression (in case of A549-IFIT1-eGFP cells) was monitored each 2–6 h in 3–5 technical replicates, each with 2–4 images per well, using a 10× magnification in an IncuCyte^®^ S3 Live-Cell Analysis System (Satorius AG, Göttingen, Germany). Incucyte® Software (2019B Rev2, Satorius AG) was used to mask cells in phase contrast images applying the following settings: segmentation adjustment 1.1, hole fill 500 µm^2^, min area filter 200 µm^2^. For green fluorescence images the following settings were applied: green acquisition time 300 ms, top-hat segmentation, radius 60 µm, threshold 1.2 gcu, edge split off, hole fill 100 µm^2^, min area filter 100 µm^2^. Cell growth was reported as phase area confluence (%) and the eGFP signal was measured as green integrated intensity per well normalized to the phase area confluence (GCU×µm^2^/well/%).

### 2.9. Total Proteome Analyses Using LC-MS/MS

NS5B-transduced A549 cells were analyzed 6, 12, 18, or 24 days post transduction, MAVS expression in HEK-FlpIn-MAVS cells was induced for 1, 8, 12, or 15 days and A549 cells were continuously treated every third day with IFN-α for 2, 6, or 14 days. For each replicate, cells were washed with PBS, lysed in SDS lysis buffer (4% SDS, 10 mM DTT, 50 mM Tris/HCl pH 7.6), boiled at 95 °C for 5 min and sonicated (4 °C, 10 min, 30 s on, 30 s off; Bioruptor, Diagenode SA, Seraing, Belgium). Protein concentrations of cleared lysates were normalized to 50 µg and cysteines were alkylated with 55 mM IAA (20 min, 25 °C, in the dark). SDS was removed by protein precipitation with 80% (*v*/*v*) acetone (−20 °C, overnight), protein pellet was washed with 80% (*v*/*v*) acetone and resuspended in 40 µL U/T buffer (6 M urea, 2 M thiourea in 10 mM HEPES, pH 8.0). Protein digestion was performed by subsequent addition of 1 µg LysC (3 h, 25 °C; FUJIFILM Wako Pure Chemical Corporation, Richmond, VA, USA) and 1 µg Trypsin (Promega, Walldorf, Germany) in 160 µL digestion buffer (50 mM ammonium bicarbonate, pH 8.0) at 25 °C overnight. Peptides were desalted and concentrated using C_18_ Stage-Tips as described previously [17]. Purified peptides were loaded onto a 50 cm reverse-phase analytical column (75 µm diameter; ReproSil-Pur C18-AQ 1.9 µm resin; Dr. Maisch, Ammerbuch-Entringen, Germany) and separated using an EASY-nLC 1200 system (Thermo Fisher Scientific) with a 120 or 180 min gradient (80% acetonitrile, 0.1% formic acid; 120 min gradient: 5–30% (90 min), 30–95% (20 min), wash out at 95% for 5 min, readjustment to 5% in 5 min; 180 min gradient: 5–30% (150 min), 30–95% (20 min), wash out at 95% for 5 min, readjustment to 5% in 5 min) at a flow rate of 300 nL per min. Eluting peptides were directly analyzed on a Q-Exactive HF mass spectrometer (Thermo Fisher Scientific). Data-dependent acquisition included repeating cycles of one MS1 full scan (300–1600 *m*/*z*, R = 60,000 at 200 *m*/*z*) at an ion target of 3 × 10^6^, followed by 15 MS2 scans of the highest abundant isolated and higher-energy collisional dissociation (HCD) fragmented peptide precursors (R = 15,000 at 200 *m*/*z*). For MS2 scans, collection of isolated peptide precursors was limited by an ion target of 1 × 10^5^ and a maximum injection time of 120 ms. Isolation and fragmentation of the same peptide precursor was eliminated by dynamic exclusion for 20 s. The isolation window of the quadrupole was set to 1.4 *m*/*z* and HCD was set to an NCE of 27% and an underfill ratio of 20%. Peptides of MAVS expressing HEK-FlpIn-MAVS cells were analyzed on a LTQ-Orbitrap XL mass spectrometer (Thermo Fisher Scientific). Proteomic analysis was performed as described earlier [18]. Briefly, data-dependent acquisition mode with one full MS1 scan at a resolution of 60,000 at 400 *m*/*z* in the Orbitrap was followed by 10 MS2 scans in the linear ion trap (target ion value = 1000, isolation width = 2 *m*/*z*, CID fragmentation mode with normalized collision energy = 40) of the highest reported peaks obtained in the full MS scan. Raw files were processed with MaxQuant (version 1.6.10.43; Cox Lab, Max-Planck-Institute of Biochemistry, Martinsried, Germany) using the standard settings, label-free quantification (LFQ) and match between run options enabled. Spectra were searched against forward and reverse sequences of the reviewed human proteome including isoforms (UniprotKB, release 10.2019) by the built-in Andromeda search engine [19].

### 2.10. Statistical Analyses

The output of MaxQuant was analyzed with Perseus (version 1.6.10.43, Tyanova, et al. [20]), R (version 3.6.0) and RStudio (version 1.2.1335). Detected protein groups identified as known contaminants, reverse sequence matches, only identified by site or quantified in less than three out of four replicates in at least one condition were excluded. Following log_2_ transformation, missing values were imputed for each replicate individually by sampling values from a normal distribution calculated from the original data distribution (width = 0.3 × s.d., downshift = −1.8 × s.d.). Differentially expressed protein groups between NS5B^WT^ and NS5B^mut^ transduced cells were identified via two-sided Student’s *t*-tests (S0 = 1) corrected for multiple hypothesis testing applying a permutation-based false discovery rate (FDR < 0.01, 250 randomizations). Protein groups were further removed for statistical testing if not at least one *t*-test condition contained a minimum of three non-imputed values. Fisher’s exact test with Benjamini–Hochberg adjusted FDR was used to perform pathway enrichment analyses of Gene Ontology terms corresponding to Biological Processes (GOBP, downloaded from http://annotations.perseus-framework.org, 06.2019) within significantly changing proteins. For hierarchical clustering with heat map representation, row-wise Z-scored log_2_ LFQ intensities were clustered using Euclidean distances and Ward as agglomeration method. Significantly upregulated proteins 6 days after NS5B^WT^ transduction (compared to NS5B^mut^) were intersected with proteins detected in IFN-α treated A549 cells or doxycycline-treated HEK-FlpIn-MAVS cells using unique identifiers for each protein group (gene name and majority protein ID). The median log_2_ LFQ intensity for each of these protein groups across replicates was calculated per condition and time point, normalized by row-wise Z-scoring and plotted using notched box and whisker plots in the style of Tukey.

### 2.11. Antibodies, Plasmids, and Primers

Antibodies, plasmids, and primers used in this study are separately listed in Supplementary File S1.

## 3. Results

### 3.1. Ligand-Based Stimulation of RIG-I by Expression of HCV NS5B

**Figure 1:**
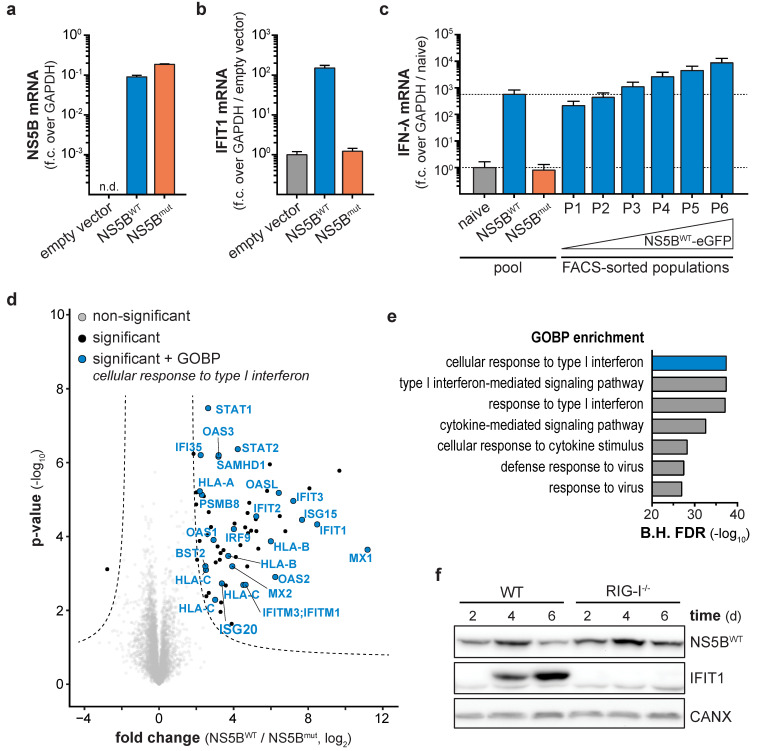
Ligand-based stimulation of retinoic acid inducible gene I (RIG-I) by expression of Hepatitis C virus (HCV) RdRP NS5B. NS5B (**a**) and IFIT1 (**b**) mRNA levels in NS5B^WT^, NS5B^mut^, or empty vector transduced A549 cells, 6 days post transduction. NS5B fold change (f.c.) over GAPDH or IFIT1 fold change over GAPDH and empty vector control was calculated (mean + s.d. of *n* = 3 technical replicates). One representative of *n* = 3 independent experiments is shown. (**c**) IFN-λ mRNA levels in naïve cells or cells transduced with NS5B^WT^ or NS5B^mut^ either correspond to the entire cell pool or FACS sub-populations (P1-6), which were sorted by increasing NS5B^WT^-eGFP levels. Fold change over GAPDH and naïve cells was calculated (mean + s.d. of *n* = 3 technical replicates). Dashed lines indicate IFIT1 mRNA levels of the cell pool for naïve or NS5B^WT^ transduced cells. (**d**) Total proteome analyses of A549 cells 12 days post NS5B^WT^ or NS5B^mut^ transduction. Hyperbolic lines indicate significantly changing proteins (two-tailed Student’s *t*-test, S0 = 1, permutation-based FDR < 0.01, *n* = 4 technical replicates). Proteins annotated with the GOBP term “cellular response to type I interferon” are highlighted in blue. (**e**) Bar plot showing Benjamini–Hochberg-adjusted (B.H.) FDR values (Fisher’s exact test) for the seven most significantly enriched GOBP terms within significantly changing proteins upon NS5B^WT^ expression. (**f**) Western blot of NS5B and IFIT1 protein expression in wild-type and RIG-I knock-out A549 cells transduced with NS5B^WT^ for the indicated days. Calnexin (CANX) was used as loading control.

Persistent virus infection generates immune-stimulatory nucleic acids as well as immune-modulatory viral proteins for a prolonged time. In contrast, synthetic stimuli commonly used to study the activity of the innate immune system in the absence of viral perturbations allow only a single pulse of stimulation. In order to continuously stimulate innate antiviral responses in a virus-free cell culture system, we used the RNA-dependent RNA polymerase (RdRP) NS5B of HCV. Based on previous reports, ectopic expression of NS5B generates aberrant RNAs capable of stimulating the nucleic acid sensor RIG-I [21,22,23]. Therefore, we generated an eGFP-tagged variant of wild-type NS5B (NS5B^WT^) as well as the catalytically inactive version NS5B^mut^ (ΔGDD or GND), both lacking the C-terminal 21 amino acids membrane anchor. Subsequently, IFN-competent A549 cells were transduced with NS5B-coding lentiviral vectors. Both NS5B variants showed comparable expression levels 6 days post transduction (Figure 1a), but only expression of NS5B^WT^ led to the induction of the interferon-induced protein with tetratricopeptide repeats 1 (IFIT1) (Figure 1b) and IFN-λ (Figure 1c). Fluorescence-activated cell sorting (FACS) revealed a dose dependency of NS5B^WT^ expression and the resulting IFN response: high NS5B^WT^-eGFP expression (population P6) induced the highest amounts of IFN-λ mRNA (≈10^4^-fold over naïve cells, Figure 1c), while cells expressing ≈10-fold less NS5B^WT^-eGFP (population P1) only led to ≈10^2^-fold induction of IFN-λ (Figure 1c, Figure S1a,b). We proceeded with a broader characterization of the NS5B-triggered response by performing total proteome analysis of NS5B^WT^ and NS5B^mut^ expressing cells (Figure 1d,e). In contrast to NS5B^mut^, NS5B^WT^ expression led to an upregulation of 63 proteins and downregulation of one protein (Figure 1d) with enriched biological processes (GOBP) clearly indicated the presence of an antiviral state in NS5B^WT^ expressing cells (Figure 1e). Among the highly expressed proteins were MX proteins, members of the IFIT family as well as ISG15 and ISG20. Induction of interferon-stimulated genes in response to NS5B^WT^ expression required RIG-I-like receptor (RLR) signaling as A549 cells with CRISPR/Cas9 targeted deletion of the cytoplasmic pathogen-recognition receptor RIG-I failed to induce IFIT1 (Figure 1f). This confirmed that ectopic expression of NS5B^WT^ leads to the production of RNA species stimulating RIG-I in a ligand-based manner even in the absence of viral genomes. We concluded that expression of NS5B^WT^ is sufficient to generate immune-stimulatory RNAs which in turn trigger a cell-intrinsic antiviral response while omitting uncontrolled interference by virus-encoded antagonists of these pathways.

### 3.2. Counter Regulation of the ISG Response Despite Continuous NS5B Expression

**Figure 2:**
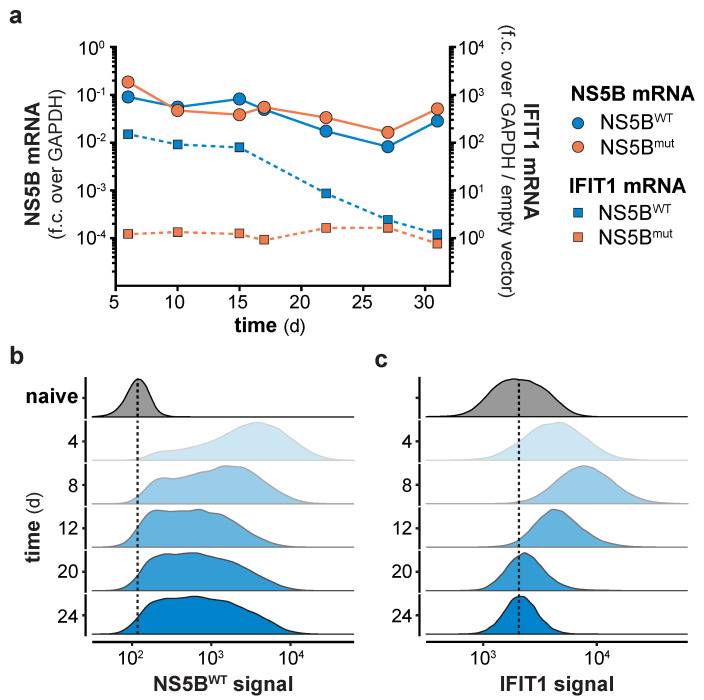
Long term NS5B expression counter-regulates the IFN-stimulated genes (ISG) response. (**a**) NS5B and IFIT1 mRNA levels in A549 cells at the indicated days post NS5B^WT^ or NS5B^mut^ transduction. NS5B fold change (f.c.) over GAPDH or IFIT1 fold change over GAPDH and empty vector control was calculated (mean ± s.d. of *n* = 3 technical replicates). One representative of *n* = 3 independent experiments is shown. NS5B-eGFP (**b**) and IFIT1 (**c**) protein levels in NS5B^WT^-eGFP transduced and naïve cells were determined by flow cytometry at the indicated days post transduction. Scaled density plots of fluorescent intensities were plotted individually for each time point after NS5B^WT^-eGFP transduction or by combining time points for naïve cells. Dashed lines highlight the median intensity in naïve cells.

**Figure 3:**
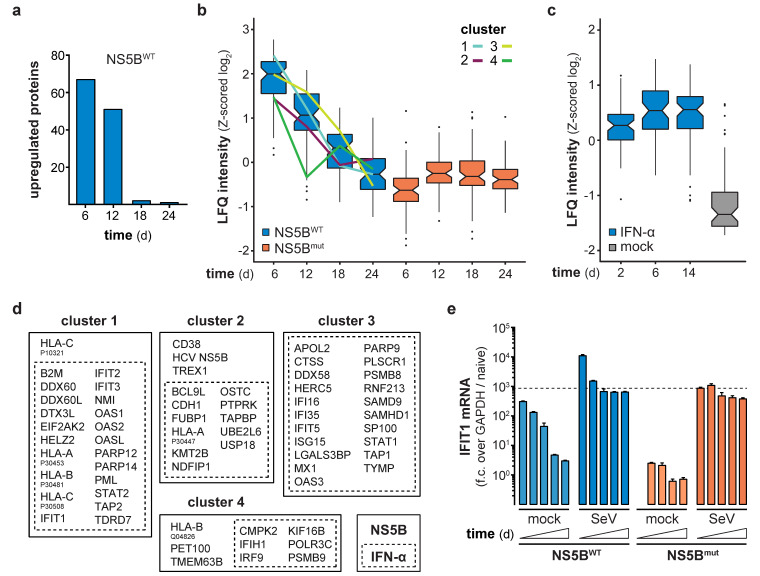
Time-resolved proteomics identifies differentially regulated proteins in NS5B and IFN-α treated cells. (**a**) Number of significantly upregulated proteins (two-tailed Student’s *t*-test, S0 = 1, permutation-based FDR < 0.01, *n* = 4 technical replicates) at the indicated days post NS5B^WT^ transduction as compared to NS5B^mut^. (**b**,**c**) Box and whisker plots (median, hinges: 1st and 3rd quartiles, whiskers: ± 1.5 × inter-quartile-range) of Z-scored median log_2_ label-free quantification (LFQ) intensities of proteins, significantly upregulated at 6 days post NS5B^WT^ transduction, tracked over time in NS5B^WT^ and NS5B^mut^ transduced (**b**) or 8.25 ng/mL IFN-α and mock treated (**c**) cells. Hierarchical clustering (Euclidean distances, Ward agglomeration method; Figure S3) of Z-scored log_2_ LFQ intensities of these proteins in NS5B^WT^ transduced cells across time points identified four main clusters of differentially upregulated proteins (cluster 1, light blue; cluster 2, purple; cluster 3, yellow; cluster 4, green). (**d**) Intersection of NS5B^WT^ (solid box) upregulated proteins at day 6 post transduction with data of IFN-α (dashed box) treated cells. Upregulated proteins were assigned to each of the identified clusters. (**e**) IFIT1 mRNA levels in A549 cells non-infected or infected with Sendai Virus (SeV) at 7, 11, 15, 19, and 23 days after NS5B^WT^ or NS5B^mut^ transduction. IFIT1 fold change (f.c., mean + s.d. of *n* = 3 technical replicates) over GAPDH and naïve cells was calculated. The dashed line indicates IFIT1 mRNA levels in SeV infected cells 7 days after NS5B^mut^ transduction.

Continuous expression of NS5B^WT^ presented an opportunity to functionally test the consequences of long-term innate immune stimulation. Previous studies indicated that constitutive expression of viral polymerases has no negative physiological impact in transgenic mice [24,25], suggesting that expression of NS5B may also be a suitable way in vitro to stimulate innate immune responses for extended periods of time. Indeed, expression of NS5B mRNA in lentivirally transduced cells was readily detected throughout the full period of observation, with the average expression levels slightly declining over time (Figure 2a). Following an initial peak of expression 4–8 days after lentiviral transduction, NS5B^WT^ protein was detected in all transduced cells and stabilized at constant levels until day 24 of the experiment (Figure 2b). As expected, NS5B^WT^ transduction induced IFIT1 expression with the highest IFIT1 protein levels at day 8 (Figure 2a,c). Surprisingly and in contrast to NS5B, IFIT1 mRNA and protein levels declined after 8 days and returned to basal levels within 3 weeks post NS5B^WT^ transduction (Figure 2a,c). In order to address if the observed shutdown of gene expression is specific to IFIT1 or if it is a general feature of the ISG response, we performed full proteome analysis of NS5B^WT^ and NS5B^mut^ transduced cells at 6, 12, 18, and 24 days post transduction. Compared to NS5B^mut^ expressing cells, we identified 67 upregulated proteins, including many prototypical ISGs, 6 days after NS5B^WT^ transduction (Figure 3a). However, the number of upregulated proteins diminished over time until day 24 (Figure 3a and Figure S2a–d). By tracking the abundance of proteins initially upregulated at day 6, we could confirm the counter regulation of the ISG response on a proteome-wide level (Figure 3b). Interestingly, time-resolved hierarchical clustering of proteins upregulated by NS5B^WT^ 6 days post transduction revealed four main clusters of differentially regulated proteins (Figure 3b,d and Figure S3). We identified proteins such as IRF9 and IFIH1 that were upregulated at day 6, but rapidly returned to base levels 12 days after transduction (cluster 4). With a similar but delayed downregulation at day 18, cluster 2 included proteins involved in antigen presentation (HLA-A, TAPBP) and ISG15 conjugation (UBE2L6, USP18). Proteins of cluster 1 showed the highest upregulation at day 6 and returned to basal levels at day 18. Interestingly, this cluster was composed of many proteins involved in nucleic acid binding (DDX60, −L, EIF2AK2, IFIT1, -2, -3, OAS1, -2, -L). A fourth cluster (cluster 3) of proteins was induced by NS5B^WT^ expression and contained negative regulators of the innate immune system including ISG15 and LGALS3BP. In general, varying expression kinetics can be explained by differences in induction sensitivities amongst ISGs, as well as different protein stabilities and half-lives. Although different kinetics of ISG expression were observed, all upregulated proteins followed the same overall trend by returning to baseline levels within 24 days despite robust NS5B^WT^ expression. 

### 3.3. Refractoriness of Type-I IFN or RLR Signaling Is Not Responsible for the ISG Counter Regulation

We asked whether the decline in ISG expression despite presence of NS5B^WT^ is inherent to the IFN system and represents its previously described refractory state [26]. Therefore, we continuously treated cells with IFN-α for 2, 6 or 14 days and analyzed total protein expression by mass spectrometry. Unlike NS5B^WT^ expression, exposure to IFN-α led to the induction of a constant number of ISGs over time (Figure 3c,d). Proteins that showed transient upregulation upon NS5B^WT^ expression were constantly induced during IFN-α treatment, indicating that these proteins are not subject to direct IFN-mediated negative feedback regulation. Overall, our data suggested that NS5B induces type-I IFNs, but these cytokines do not lead to an anergic state rendering cells unresponsive to further stimulation. Having ruled out type-I IFN refractoriness as a cause for the observed downregulation of ISGs upon prolonged RIG-I-stimulation, we considered further scenarios that could explain a reduced antiviral response over time. First, it may not be IFN signaling but rather the viral nucleic acid sensing pathway leading to IFN induction that becomes refractory upon prolonged activation. In order to test this hypothesis, we assessed whether NS5B expressing cells were still capable of properly responding to viral infection. To this end, cells were infected with Sendai virus (SeV) at various days after NS5B^WT^ or NS5B^mut^ transduction and IFIT1 expression was measured (Figure 3e). As expected, NS5B^WT^ transduced cells elicited IFIT1 transcription early after transduction also in the absence of SeV infection, and this response declined over time. NS5B^mut^ expressing cells did not show a priori upregulation of IFIT1, but could be stimulated by SeV infection to generate IFIT1 levels comparable to those observed in NS5B^WT^ cells. Interestingly, NS5B^WT^ expressing cells could be further stimulated by SeV infection, yielding maximum IFIT1 levels at all tested time points (Figure 3e). This indicates that constitutive stimulation of the RLR pathway by NS5B does not lead to a general state of unresponsiveness of the antiviral system. 

### 3.4. NS5B Expression but Not IFN Treatment Leads to Reduced Cell Growth

**Figure 4:**
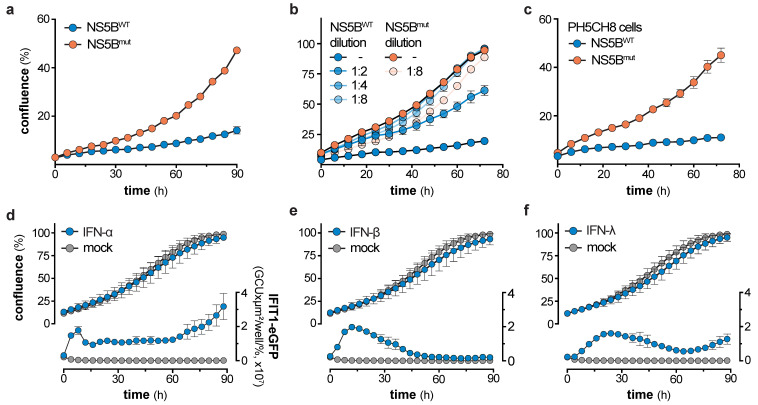
NS5B expression but not IFN treatment leads to reduced cell growth. (**a**) Cell growth of A549 cells 5 days after NS5B^WT^ or NS5B^mut^ transduction as determined by live cell imaging (mean ± s.d. of *n* = 5 technical replicates). One representative of *n* = 3 independent experiments is shown. (**b**) Cell growth of A549 cells 5 days after NS5B^WT^ or NS5B^mut^ transduction with increasing dilutions of NS5B expressing lentivirus (mean ± s.d. of *n* = 4 technical replicates). (**c**) Cell growth of PH5CH8 cells 5 days after NS5B^WT^ or NS5B^mut^ transduction as determined by live cell imaging (mean ± s.d. of *n* = 4 technical replicates). One representative of *n* = 3 independent experiments is shown. (**d**,**f**) Cell growth of A549-IFIT1-eGFP cells treated with 8.25 ng/mL IFN-α (**d**), 17 IU/mL IFN-β (**e**), or 9 ng/mL IFN-λ (**f**) for the indicated time (mean ± s.d. of *n* = 4 technical replicates and *n* = 3 independent experiments). Left y-axis shows the percentage confluence and the right y-axis the IFIT1-eGFP expression based on the confluence-normalized green integrated intensity per well.

Loss of ISG expression over time in NS5B^WT^ expressing cells was neither due to refractoriness of the IFN system (Figure 3a–d), nor did long-term NS5B^WT^ expression render the RLR pathway anergic towards virus infection (Figure 3e). Knowing that stably transduced cells exhibit a broad range of NS5B^WT^ protein levels (Figure 2b) directly correlating with the magnitude of IFN induction (Figure 1c and Figure S1), we hypothesized that cells expressing the highest levels of NS5B^WT^ might suffer a viability or growth deficit and hence get counter selected over time. Indeed, live cell imaging revealed a significant and dose-dependent impact of NS5B^WT^ expression on cell growth (Figure 4a–c and Figure S4a–c). Interestingly, this phenotype was only present for the first days after transduction, where both, NS5B^WT^ expression and IFIT1 induction were strongly elevated (Figure 2b,c). We therefore analyzed cell growth in this period from 5 to 9 days post transduction of A549 cells and confirmed our findings in human hepatocyte-derived PH5CH8 cells. Proliferation rates of NS5B^WT^ transduced cells were ≈3-times lower as compared to NS5B^mut^ transduced control cells and directly correlated with NS5B expression levels (Figure 4a–c). This clear cell growth disadvantage over cells not expressing active NS5B likely led to a gradual depletion of cells expressing NS5B^WT^ at levels sufficient to induce production and secretion of type-I IFNs. It has previously been reported that type-I IFNs can exhibit cytotoxic and cytostatic effects [27,28,29], which could account for the observed growth defect of cells expressing highest levels of NS5B^WT^. To test a potential involvement of IFN-α in our system, we treated an A549-IFIT1-eGFP reporter cell line with high doses of IFN-α and tested for cell proliferation (Figure 4d). This cell line allowed us to simultaneously track cell growth and ISG induction (IFIT1-eGFP expression) over time. Notably, high doses of IFN-α induced prominent and sustained IFIT1 induction, indicative of active type-I IFN signaling. However, IFN-α treatment did not affect cell growth of A549 cells, which was further true for IFN-β and -λ (Figure 4d–f). IFN-β and -λ were used at concentrations that induced IFIT1 expression levels comparable to treatment with IFN-α, thereby excluding IFN-subtype specific effects on cell growth. Hence, our data indicates that type-I and -III IFN signaling is efficacious even when supplied for prolonged periods of time, but IFN signaling is not responsible for the observed growth defect of NS5B^WT^ expressing cells. These observations are in line with the proteomic analysis of cells exposed to IFN-α for 14 days (Figure 3c,d).

### 3.5. MAVS- and IRF3-Dependent Counter Selection of NS5B Expressing Cells

**Figure 5:**
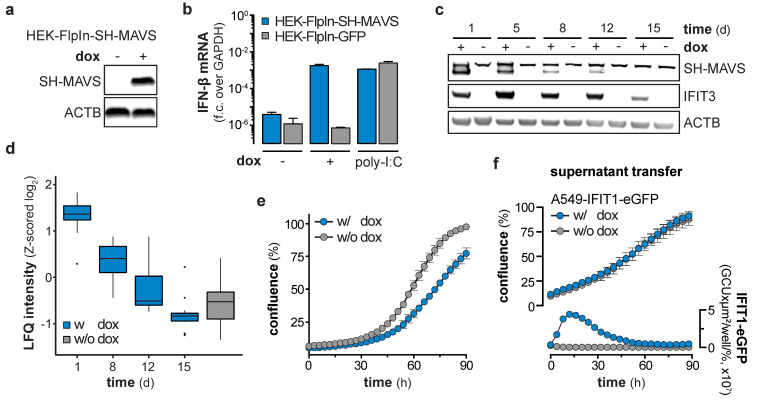
Counter selection of mitochondrial antiviral signaling protein (MAVS) expressing cells. (**a**) Western blot showing protein expression levels of StrepII-HA-tagged MAVS (SH-MAVS) and β-actin(ACTB) in doxycycline (dox)-treated HEK-FlpIn-MAVS cells. (**b**) IFN-β mRNA levels in HEK-FlpIn-GFP or -SH-MAVS cells, non-treated, 1 µg/mL dox-treated or poly-I:C (1 µg / 1 × 10^6^ cells) transfected for 1 day. Fold change (f.c.) over GAPDH was calculated (mean + s.d. of *n* = 2 technical replicates). (**c**) Western blot of SH-MAVS, IFIT3, and ACTB protein expression in HEK-FlpIn-MAVS cells non-treated or continuously treated with 1 µg/mL dox for the indicated days. (**d**) Total proteome analysis of HEK-FlpIn-MAVS cells non-treated or 1 µg/mL dox-treated for the indicated days. Box and whisker plots (median, hinges: 1st and 3rd quartiles, whiskers: ± 1.5 × inter-quartile-range) represent row-wise Z-scored median log_2_ LFQ intensities (*n* = 4 technical replicates) of proteins, overlapping with proteins significantly upregulated at 6 days post NS5B^WT^ transduction. (**e**) Cell growth assay of HEK-FlpIn-MAVS cells non-treated or 1 µg/mL dox-treated for the indicated hours (mean ± s.d. of *n* = 9 technical replicates). One representative of *n* = 3 independent experiments is shown. (**f**) Cell growth and IFIT1-eGFP expression of A549-IFIT1-eGFP cells stimulated with the supernatant of 1 day 1 µg/µL dox-treated HEK-FlpIn-MAVS cells (mean ± s.d. of *n* = 4 technical replicates of *n* = 3 independent experiments). Left y-axis shows the percentage confluence and the right y-axis the IFIT1-eGFP expression based on the confluence-normalized green integrated intensity per well.

**Figure 6:**
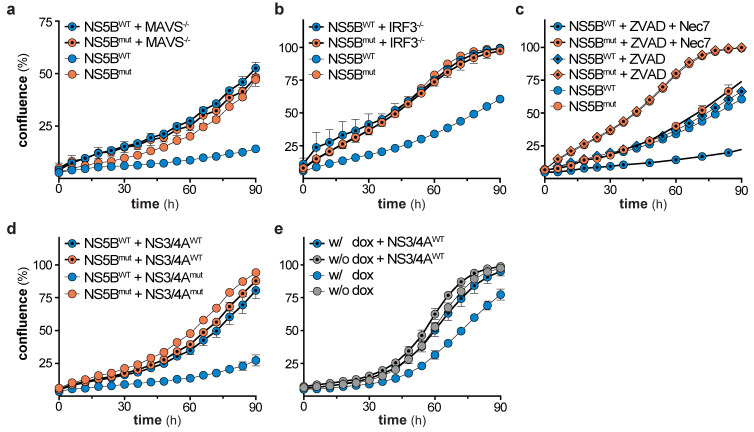
Mitochondrial antiviral signaling protein (MAVS)- and interferon regulatory factor 3 (IRF3)-dependent negative selection of NS5B expressing cells and rescue by HCV NS3/4A. (**a**) Cell growth of wild-type and MAVS knock-out A549 cells, 5 days after transduction of either NS5B^WT^ or NS5B^mut^ (mean ± s.d. of *n* = 5 technical replicates). One representative of *n* = 3 independent experiments is shown. (**b**) Cell growth of wild-type and IRF3 knock-out A549 cells, 5 days after transduction of either NS5B^WT^ or NS5B^mut^ (mean ± s.d. of *n* = 5 technical replicates). One representative of *n* = 3 independent experiments is shown. (**c**) Cell growth of DMSO, Z-VAD-FMK (ZVAD, 40 µM), or Necrostatin-7 (Nec7, 10 µM) treated A549 cells, 5 days after transduction of either NS5B^WT^ or NS5B^mut^ (mean ± s.d. of n = 4 technical replicates). One representative of *n* = 2 independent experiments is shown. (**d**) Cell growth of NS5B^WT^ or NS5B^mut^ and NS3/4A^WT^ or NS3/4A^mut^ transduced A549 cells (mean ± s.d. of *n* = 3 technical replicates). One representative of *n* = 3 independent experiments is shown. (**e**) Cell growth of non-treated or dox-treated HEK-FlpIn-MAVS cells, 5 days after NS3/4A^WT^ or NS3/4A^mut^ transduction (mean ± s.d. of *n* = 9–12 technical replicates). One representative of *n* = 3 independent experiments is shown.

We showed that NS5B triggers the IFN system via the RIG-I/MAVS/IRF3 axis (Figure 1f), but IFN signaling was not responsible for the observed reduction in cell growth (Figure 4). Therefore, we concluded that the cause for the growth retardation and ceasing of ISG expression has to be upstream of IFN production, likely at or downstream of the central adapter protein MAVS. Hence, we tested if the ISG response in cells stimulated by overexpression of MAVS (independent of RIG-I) shows the same decline upon long-term stimulation. We generated HEK-FlpIn-MAVS cells that allowed for doxycycline (dox)-controlled induction of StrepII-HA (SH)-tagged MAVS (Figure 5a). It has previously been reported that overexpression of this signaling adapter triggers its RIG-I-independent oligomerization and strong activation of downstream signaling [30]. As expected, dox-treated HEK-FlpIn-MAVS cells generated IFN-β mRNA to an extent similar to poly-I:C-stimulated control cells (Figure 5b). Interestingly, SH-MAVS expression could only be observed up to day 12 of continuous dox treatment (Figure 5c). Expression of MAVS was accompanied by strong induction of IFIT3, which slowly decreased over the 15 day course of the experiment (Figure 5c), reminiscent of the ISG counter regulation upon prolonged NS5B expression. We again employed proteomics to confirm that the effect observed for IFIT3 was true for the global ISG response, and whether the proteomic expression pattern is comparable to the expression pattern observed in NS5B^WT^ transduced cells. Indeed, proteins upregulated by NS5B^WT^ were also induced by overexpression of MAVS (e.g., IFIT1, -2, -3, STAT1, ISG15), but gradually disappeared within 15 days despite continuous dox treatment (Figure 5d). When MAVS expression was upheld for several days, cells revealed a significantly reduced growth rate (Figure 5e) which was in line with NS5B^WT^ expressing cells and the notion that strong MAVS activity can induce apoptosis [31,32,33,34]. This highly similar decline in ISG expression and growth reduction of MAVS expressing cells led us to assume that in both systems a counter selection against the most strongly stimulated cells occurred. To test whether the effect on cell growth in the MAVS expressing system was cell-intrinsic or mediated through soluble factors, we expressed MAVS for 1 day in HEK-FlpIn-MAVS cells and transferred the supernatant to A549-IFIT1-eGFP reporter cells. As expected, the supernatant of dox-treated HEK-FlpIn-MAVS cells induced a robust IFN response (Figure 5f). However, this treatment did not affect cell growth. Collectively, these experiments show that MAVS expression, similar to NS5B^WT^, affects cell growth and is not capable of maintaining a stable ISG response over a course of 2–3 weeks. The phenotype observed in MAVS expressing cells was highly reminiscent of the phenotype seen when overexpressing NS5B^WT^. We therefore tested if MAVS depletion in NS5B^WT^ expressing cells could alleviate the negative effect of NS5B^WT^ on cell growth. Notably, CRISPR/Cas9 targeted deletion of MAVS in A549 cells indeed reversed the growth disadvantage (Figure 6a). While MAVS has been shown to serve as signaling hub for a variety of pathways [30,35], the induction of antiviral genes requires the transcription factor IRF3. To reveal a potential involvement of IRF3 in the NS5B^WT^ mediated cell growth phenotype we used CRISPR/Cas9 based IRF3 knock-out cells (Figure S4d). In line with our hypothesis, depletion of IRF3 rescued the NS5B mediated cell growth arrest (Figure 6b). Activation of the RLR signaling pathway has previously been suggested to lead to a MAVS- or IRF3-regulated cell death through various pathways [31,36,37,38]. However, in our system we did not observe phenotypic signs of cell death. Moreover, treatment of cells with the pan-caspase inhibitor Z-VAD-FMK (ZVAD) only or together with the necroptosis inhibitor Necrostatin-7 (Nec-7) did not rescue the NS5B^WT^ induced cell growth defect (Figure 6c). This indicates that MAVS triggers IRF3-dependent signaling events or expression of effector genes which are responsible for the observed caspase-independent cytostasis.

### 3.6. Rescue of Cell Growth through Viral Counter Measures

Based on above results, we concluded that continuous stimulation of the RLR pathway leads to a MAVS- and IRF3-dependent, but IFN-independent counter selection of cells. Accordingly, persistent viruses need to shutdown RLR signaling not only to prevent recognition and subsequent induction of IFN signaling, but also to escape metabolic disadvantage and counter selection of cells with activated RLR pathways. HCV, for example, encodes the protease NS3/4A that cleaves MAVS and thereby blunts signal transduction [7]. To test if NS3/4A is indeed able to reverse the observed growth disadvantage, we co-transduced NS5B^WT^ with NS3/4A^WT^ and tested for cell growth as well as IFIT1 induction (Figure 6d and Figure S4e). In fact, expression of NS3/4A^WT^ restored cell growth rates to levels seen for NS5B^mut^ control cells (Figure 6d). NS5B^WT^ expressing cells co-transduced with the enzymatically inactive mutant NS3/4A^mut^ continued to exhibit a strong defect in cell growth (Figure 6d). To corroborate these findings, we used HEK-FlpIn-MAVS cells transduced with NS3/4A^WT^. As for RIG-I-stimulation by NS5B^WT^ expression, MAVS overexpression led to a growth disadvantage of cells which could be rescued by NS3/4A^WT^ expression (Figure 6e). 

**Figure 7:**
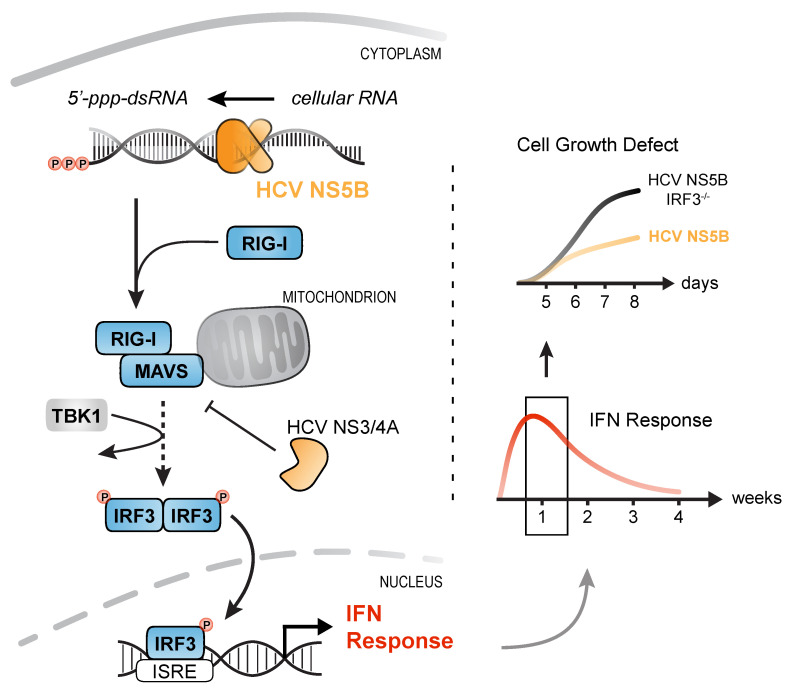
IRF3-mediated, but interferon- and caspase-independent cytostasis upon prolonged activation of RLR signaling. Expression of HCV NS5B generates virus-like immune-stimulatory dsRNAs from cellular RNA that are detected by the pattern-recognition receptor RIG-I. Activation of RIG-I triggers downstream signaling via MAVS, IRF3 phosphorylation, and subsequent translocation into the nucleus. IRF3 binding to the IFN-β and -λ promotor enhancers induces expression of IFNs which activate the antiviral immune system. Prolonged stimulation of the innate immune response caused a prominent IRF3-mediated growth defect of cells most strongly stimulated and was independent of IFN signaling or caspase activity. By targeting MAVS through NS3/4A, HCV not only dampens activation of the IFN response, but also restores cell viability in infected cells.

In summary, we show that expression of an RNA virus RdRP (HCV NS5B) leads to aberrant, putatively randomly primed synthesis of RNAs that are recognized by the innate immune sensor RIG-I. We could show that stable transduction of cells with NS5B^WT^ is an elegant means of triggering the RLR pathway at the ligand level, omitting manipulation of the pathway itself. Surprisingly, the elicited antiviral ISG response ceased over the course of 2–3 weeks due to growth deficits of strongly stimulated cells. We could demonstrate that this cytostatic effect is not mediated by IFNs or caspase-dependent apoptosis, but is regulated by RLR signaling involving MAVS as well as IRF3. This indicates that viruses that continuously trigger the RLR pathway do not only have to deal with the ensuing antiviral effector state, but also with reduced viability of their host cells. Hence, by targeting MAVS through the NS3/4A protease, HCV reduces production of IFNs and in the same turn restores cell viability (Figure 7).

## 4. Discussions

Despite potent measures of the innate and adaptive immune system, some viruses are able to establish a persistent infection. While viruses such as Hepatitis B virus follow a stealth strategy that prevents detection by the immune system [39,40], others, such as HCV, constantly trigger antiviral signaling [41]. Continuous stimulation of the innate immune system has been linked to autoimmune diseases, cancer, and tissue damage [42,43,44,45,46]. Moreover, accumulating evidence underlines the existence and importance of negative regulation of antimicrobial immune responses, which would affect clearance of viral pathogens [6]. Hence, studying the effects of prolonged activation of antiviral pathways is crucial to understand the molecular mechanisms leading to chronicity.

In the present study, we developed an experimental system allowing for continuous ligand-based stimulation of the cell-intrinsic antiviral signaling pathway in absence of any perturbation, e.g., overexpression of pathway components or virus-encoded antagonists. For this purpose, we ectopically expressed the RdRP of HCV, which has previously been shown to give rise to immune-stimulatory RNA products inducing a prototypical antiviral ISG response. While an early report described antiviral signaling upon NS5B expression to be mediated through (overexpressed) TLR3 [29], more recent studies clearly attributed ISG production to activation of the RLR/MAVS axis [21,23]. In line with these reports, we observed a strict dependence of ISG expression on the presence of RIG-I and MAVS. Moreover, HCV infection was reported to generate an almost exclusively RLR-dependent signal and actively evades TLR3-sensing by secreting double-stranded RNA intermediates through the exosomal pathway [47]. Apart from RIG-I and TLR3, Vegna et al. [48] discovered NOD1 as an additional cytosolic receptor that, together with its signaling adaptor RIPK2, induces expression of ERK, IL-8, and TNF-α upon NS5B activity. In accordance with our results, they also found an involvement of RIG-I in IFN-β induction upon NS5B expression and could rule out that the two pathways were coupled. 

Consequently, stable transduction of cells with NS5B promised to be a suitable system to continuously stimulate the RIG-I pathway by constantly supplying intracellular RNA ligands. Surprisingly, ISG expression reproducibly faded approx. 1 week after transduction, and completely ceased after 2–3 weeks, despite ongoing NS5B expression. Such a dampening of the observed ISG signature could be due to activation of negative feedback regulations. Interferon stimulated proteins such as USP18, SOCS1/2, DAPK1, ISG15, LGALS3BP [12,13,49,50,51,52], and others have been shown to negatively regulate IFN induction and/or IFN signaling. However, proteomic analyses did not show long-term upregulation of negative regulators, but only short-term induction (e.g., of ISG15 and LGALS3BP) and subsequent downregulation, comparable to the global ISG response upon prolonged NS5B expression. Moreover, challenging of NS5B expressing cells with RIG-I inducing Sendai virus yielded robust ISG induction at every time point, strongly arguing against an induction of negative feedback loops rendering cells refractory to IFN signaling and ISG production. Detection of NS5B generated immune-stimulatory RNAs throughout the experiment was further attempted by total RNA isolation and treatment of reporter cells to monitor RNA levels capable of inducing RLR signaling. This approach failed, indicating that the fraction of stimulatory RNA within the total isolated RNA was too little to induce a detectable reporter activation. Strong dilution of the immune-stimulatory RNA was likely caused by the small number of cells expressing high levels of NS5B versus the large number of NS5B low expressing cells. We have further performed experiments employing continuous treatment of cells with IFN-α for 2 weeks, but did not observe a downregulation of ISGs. This is in contrast to the concept of IFN refractoriness [26,53], which, however, was mostly described in mice and might be strongly cell-type specific.

While the decrease in strength of the innate immune response over time did not appear to be due to an active suppression by negative immune regulators, it correlated with a gradual reduction of NS5B expression, which stabilized at lower levels. This suggests a type of negative regulation that only permits high level expression of NS5B for a short period of time. A possible explanation for reduced NS5B expression in a cell population could be negative selection of cells that express high amounts of NS5B and, as a result, induce a strong antiviral response. Indeed, NS5B expression had a significant cytostatic effect, but the underlying reason was not clear. It is widely accepted that high amounts of type-I IFNs have cytostatic and/or cytotoxic effects [27,28,29]. However, prolonged treatment with recombinant IFN-α, -β or -λ did not affect cell proliferation in our system. Therefore, it appeared likely that the observed impact on cell growth was mediated by the upstream IFN-inducing pathways. We showed that NS5B expression activates RIG-I- and MAVS-dependent signaling, suggesting that this pathway may be involved in reduced cell proliferation. Indeed, RIG-I activation has been linked to cell death induction [33,54] and MAVS transfection (i.e., auto-activation) activates apoptosis [32]. In line with an involvement of this pathway in the observed impairment of cell growth, CRISPR/Cas9 mediated deletion of MAVS restored normal growth in the presence of high level NS5B expression. This effect was remarkable since it suggests that other pattern recognition receptors, such as protein kinase R, Toll-like receptor 3 or the 2′5′oligoadenylate synthetase/RNAseL system, all of which are activated by double-stranded RNA and linked to cell death induction [55,56,57], appear to not or only marginally regulate cell growth upon long-term NS5B expression. Furthermore, manipulation of the pathway downstream of MAVS by deletion of the transcription factor IRF3 restored normal cell growth despite NS5B activity. Recently, a RIG-I-like receptor-induced IRF3 mediated pathway of apoptosis (RIPA) has been described [37,38,58]. Here, linear poly-ubiquitination of IRF3 through the LUBAC complex involving, among others, MAVS and TRAF2/6 facilitates the recruitment of IRF3 and BAX to the mitochondrial membrane, subsequent release of pro-apoptotic factors such as cytochrome C, and activation of caspase 9. Induction of RIPA through NS5B-derived dsRNA posed a straightforward explanation for the observed growth defect in NS5B transduced cells. However, in our system, we did not observe signs of cell death that could explain the strong growth retardation. Additionally, treatment with the pan-caspase inhibitor Z-VAD-FMK had no effect on cell growth, excluding a major involvement of caspase-driven apoptosis. This suggests the presence of a RIPA-independent cytostatic property of IRF3, which might be linked to transcriptional activity. Previous in vitro studies on tumor cells also indicated an oncogenic potential upon knock-out or mutation of IRF3, while overexpression decreased cell growth and cell cycle progression, partly through blocking DNA synthesis and apoptosis induction [59]. In particular, induction of apoptosis was found to be interferon-independent and linked to the transcriptional activity of DRAF1, a dsRNA-activated transcription factor complex composed of IRF3 and CREBBP [60]. These studies are in line with our findings of an interferon-independent, but IRF3-activated cytostatic effect upon stimulation of RLR signaling by NS5B activity. Since IRF3 does not only lead to pro-inflammatory gene expression but has also been linked to induction of negative regulators [61,62,63], the here discovered function may be part of this transcriptional program.

Combining our observations, we conclude that—at least in immune-competent A549 cells—it is not possible to induce a constitutive antiviral state through triggering the RLR pathway. Initial stimulation of RIG-I in NS5B high-expressing cells led to the production of type-I and -III IFNs that communicated the antiviral signal across the whole cell population. On the one hand, as RLRs themselves are ISGs, this IFN response in a positive feedback could further increase the cells’ sensitivity towards NS5B-generated RNA, thereby rendering the response strong and robust. On the other hand, NS5B expression gradually decreased throughout the cell population which can be associated with a clear cell growth deficit of cells with strong RLR activation, leading to a steady counter selection against IFN producing cells. The combination of both effects, IFN-mediated upregulation of RLR-sensitivity and constant loss of the inducing stimulus, is a plausible explanation for the observed robust and stable expression of ISGs for a certain period of time, followed by a steep decline to background levels as soon as NS5B levels drop below a certain threshold. The here presented cell culture model is therefore well-suited to study the effects of persistent ligand-based stimulation of innate antiviral pathways without viral interference. This model system can serve as a basis to broaden our understanding of the prerequisites for a virus to achieve persistence in the face of a functional cell-intrinsic immune response.

It is intriguing to envisage how the process described above—increasing both, sensitivity to viral nucleic acids and cellular countermeasures against their amplification—acts during viral infections in a tissue context. High-level viral replication in a cell is accompanied by PAMP production and, hence, leads to secretion of IFNs into the surrounding tissue region. While this fortifies antiviral responses through upregulation of RLRs, the infected cells would suffer from the cytostatic/metabolic burden of prolonged RLR-signaling as demonstrated in our experiments. Eventually, this would efficiently limit viral spread and replication, suppress production of PAMPs, and ultimately lead to a gradual ceasing of the antiviral response. This implies that it is very difficult for RLR-stimulatory viruses to persistently replicate in a cell. Indeed, HCV, a virus that frequently establishes persistent infections, has evolved several strategies to counteract those measures. Strategies include PAMP level reduction through self-limiting RNA replication and active export of dsRNA species [47,64]. Furthermore, while the infection is persistent on the organ level, it was reported to be highly dynamic on a single cell level [10,65,66]. Livers infected with HCV contain small clusters of infected cells that are surrounded by non-infected tissue [65,66]. Both, infected and non-infected regions, exhibit elevated ISG levels [10]. In line with our model, these infected clusters appear to be highly dynamic and are estimated to rarely be older than 1 week [66]. These dynamics support the notion that RLR stimulation is timely limited, and in order to establish and maintain persistence, HCV has to continuously re-infect new cells in tissue areas not currently engaged in an antiviral state. Lastly, HCV’s NS3/4A protease targets MAVS as central player in the RLR pathway to dampen the IFN-mediated immune response and IRF3-dependent apoptosis [8,58]. The here presented data propose that targeting MAVS might be crucial for establishing persistence as it relieves the cytostatic effects induced by RLR signaling, adding yet another layer of antiviral properties to this pathway.

Collectively, our data indicate that persistent activation of the RIG-I signaling pathway is counter selected, which could serve as a cellular strategy to prevent persistent virus infection. RLR activating viruses either have to reduce PAMP production or actively prevent IRF3 activation. Our work suggests a rationale for persistent viruses to directly target the RLR signaling pathway that leads to IRF3 activation since the RLR pathway—besides being responsible for expressing antiviral cytokines—initiates cytostatic effects that result in counter selection of persistently infected cells.

## Supplementary Materials

The following are available online at https://www.mdpi.com/1999-4915/12/6/635/s1, Figure S1. Fluorescence-activated cell sorting of NS5B^WT^-eGFP transduced A549 cells. Figure S2. Total cell proteomics of NS5B-transduced A549 cells. Figure S3. Hierarchical clustering of NS5B^WT^ upregulated proteins. Figure S4. NS5B and IFN-λ mRNA levels in A549 and PH5CH8 cells; Western blot of IRF3 protein expression in A549 IRF3 knock-out cells; flow cytometric analysis of IFIT1 protein expression in NS5B and NS3/4A transduced A549 cells. File S1. Antibodies, plasmids, and primers. File S2. Proteomics raw data.
