# Supplementary material for: Persistent Innate Immune Stimulation Results in IRF3-Mediated but Caspase-Independent Cytostasis
Source: Viruses. 2020 Jun 11;12(6):635. doi: 10.3390/v12060635 (PMC7354422; doi:10.3390/v12060635)
Supplement: Supplementary file 1 [file viruses-12-00635-s001.zip › Supplementary Figures S1-4.pdf]

viruses

Supplementary Material

Supplementary Figures S1-4

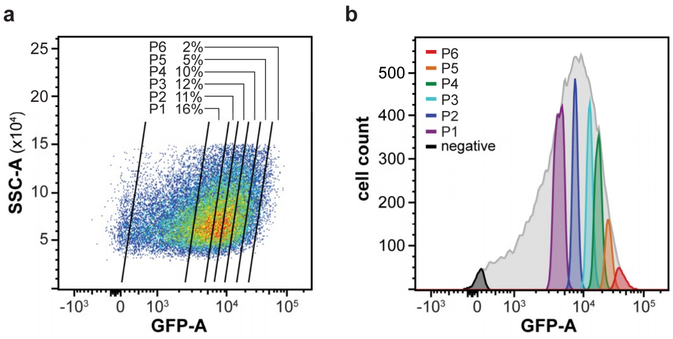

Figure S1. Fluorescence-activated cell sorting of $NS5B^{WT}$-eGFP transduced A549 cells. (**a**) Scatter plot showing the side scatter area (SSC-A) and eGFP fluorescence intensity area (GFP-A) 6 days after $NS5B^{WT}$-eGFP transduction. Non expressing (negative) cells or six populations of cells (P1-6) expressing increasing $NS5B^{WT}$-eGFP levels are separated by vertical lines. The total percentage of cells for each sub-population is indicated. (**b**) Histograms show the GFP-A for negative (black) and $NS5B^{WT}$-eGFP expressing sub-populations.

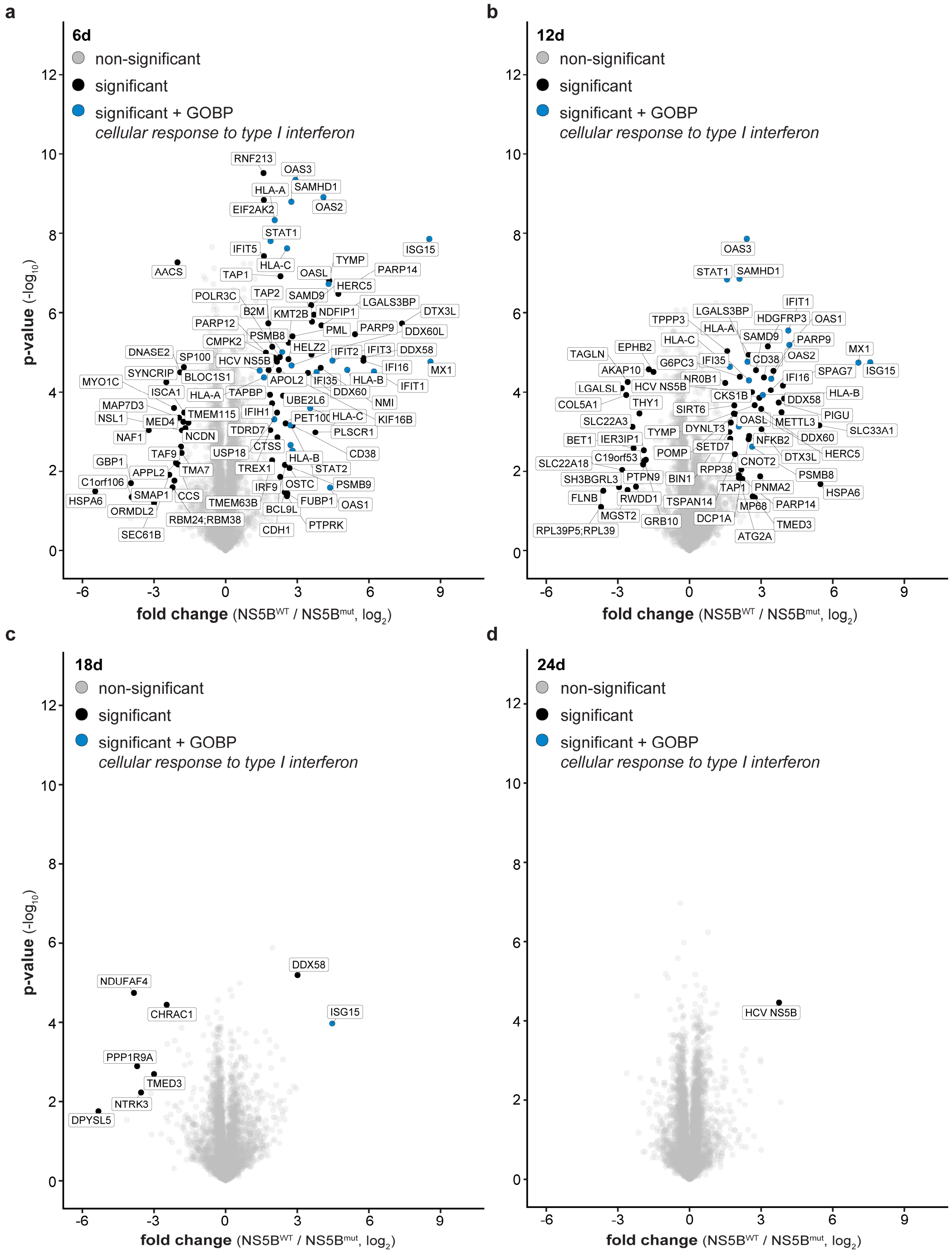

Figure S2. Total cell proteomics of NS5B-transduced A549 cells. Total cell proteomics of A549 cells 6 (**a**), 12 (**b**), 18 (**c**) and 24 (**d**) days post transduction of NS5B^{WT} versus NS5B^{mut}. Significantly up- or down-regulated proteins (two-tailed Student's T-test, S0 = 1, permutation-based FDR < 0.01, n=4 technical replicates) are shown in black and proteins annotated with the GOBP term "cellular response to type I interferon" are highlighted in blue.

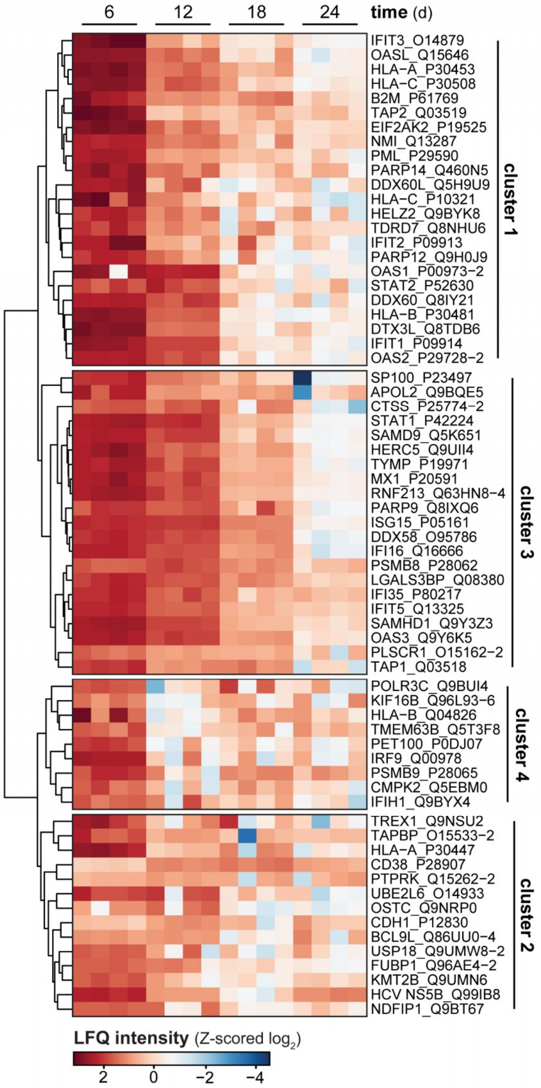

Figure S3. Hierarchical clustering of NS5B^{WT} upregulated proteins. Hierarchical clustering of Z-scored log_2 LFQ intensities of proteins, significantly upregulated 6 days after NS5B^{WT} as compared to NS5B^{mut} transduced cells. Clustering was performed for all time points after NS5B^{WT} transduction using Euclidean distances and Ward as agglomeration method. Four main clusters of differentially regulated proteins were identified and gene names including majority protein IDs for each protein are shown.

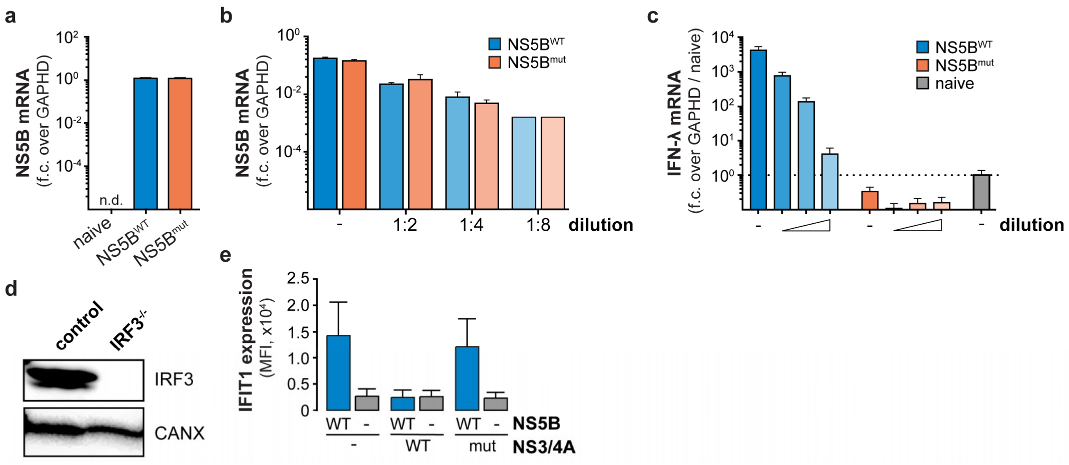

Figure S4. (**a**) NS5B mRNA levels in naïve, NS5B^{WT} or NS5B^{mut} transduced PH5CH8 cells, 5 days post transduction. NS5B fold change (f.c.) over GAPDH was calculated (mean + s.d. of n=3 technical replicates). NS5B (**b**) and IFN-λ (**c**) mRNA levels in naïve, NS5B^{WT} or NS5B^{mut} transduced A549 cells, 5 days post transduction using indicated dilutions of NS5B expressing lentiviruses. NS5B fold change over GAPDH and IFN-λ fold change over GAPDH and naïve control was calculated (mean + s.d. of n=3 technical replicates). (**d**) Western blot of IRF3 protein expression in control and IRF3 knock-out A549 cells. Calnexin (CANX) was used as loading control. (**e**) Bar graph shows the mean fluorescence intensity (MFI) of IFIT1 in A549 cells transduced as indicated with NS5B^{WT} and/or NS3/4A^{WT} or NS3/4A^{mut} (mean + s.d. of n > 1.6×10^4 single cell events). One representative of n=2 independent experiments is shown.
