# Supplementary material for: Persistent Innate Immune Stimulation Results in IRF3-Mediated but Caspase-Independent Cytostasis
Source: Viruses. 2020 Jun 11;12(6):635. doi: 10.3390/v12060635 (PMC7354422; doi:10.3390/v12060635)
Supplement: Supplementary file 1 [file viruses-12-00635-s001.zip › Supplementary File S1.pdf]

viruses

MDPI

Supplementary Material

Supplementary File S1

Table S1.1. Plasmids

Name	Origin
pDONR207	Invitrogen, Carlsbad, CA
pCMV-dr8.91	Didier Trono, EPFL, Lausanne, Schweiz
pMD2.G	Didier Trono, EPFL, Lausanne, Schweiz
pWPI BLR rfB	Binder *et al.*, 2007
pWPI EF1a BLR Con1 NS3/4A wt	Volker Lohmann, Heidelberg University Hospital, Germany
pWPI EF1a BLR Con1 NS3/4A S139A	Volker Lohmann, Heidelberg University Hospital, Germany
lentiCRISPRv2 puro	Feng Zhang, Addgene #52961
pWPI EF1a BLR NS5B dc21 WT eGFP	This study
pWPI EF1a BLR NS5B dc21 dGDD eGFP	This study
pWPI EF1a BLR NS5B dc21 GND eGFP	This study
lentiCRISPRv2 puro IRF3 guideRNA 1	This study

Table S1.2. Primers

Name	Purpose	Sequence
GAPDH fwd	qPCR GAPDH	TCGGAGTCAACGGATTTGGT
GAPDH rev	qPCR GAPDH	TTCCCGTTCTCAGCCTTGAC
ISG56 fwd	qPCR IFIT1	GAATAGCCAGATCTCAGAGGAGC
ISG56 rev	qPCR IFIT1	CCATTTGTACTCATGGTTGCTGT
IFN-λ-1 fwd	qPCR IFN-λ	GGTGACTTTGGTGCTAGGCT
IFN-λ-1 rev	qPCR IFN-λ	TGAGTGACTCTTCCAAGGCG
IFN-β-1 fwd	qPCR IFN-β	CATTCGGAAATGTCAGGAGC
IFN-β-1 rev	qPCR IFN-β	TGGAGCATCTCTTGGATGG
NS5B d21 fwd	qPCR NS5B	CAAGTATGGATTCGGGGCCA
NS5B d21 fwd	qPCR NS5B	CAGGGTAAACGATGAGGCGA
attB1NS5B fwd	PCR	GGGGACAAGTTTGTACAAAAAAGCAGGCTTCATGTC ATACTCCTGGACCGG
NS5B_GFP fwd	PCR	CGCGCGCCCGACCCCGCGTGAGCAAGGGCGAGG
NS5B_GFP rev	PCR	CCTCGCCCTTGCTCACGCGGGGTCGGGCGCG
attB2GFP rev	PCR	GGGGACCACTTTGTACAAGAAAGCTGGGTTTTACTTG TACAGCTCGTCC

Table S1.3. Antibodies

Name	Origin	Identifier
Donkey polyclonal anti-Rabbit Alexa Fluor 647	Invitrogen, Carlsbad, CA	A-31573
Goat polyclonal anti-mouse-HRP	Sigma Aldrich, Munich, Germany	A4416
Goat polyclonal anti-rabbit-HRP	Sigma Aldrich	A6154
Mouse monoclonal anti-ACTB	Santa Cruz, Dallas, TX	SC-47778
Rabbit monoclonal anti-HA-HRP	Cell Signaling, Danvers, MA	3724
Rabbit monoclonal anti-IRF3 (D6I4C) XP	Cell Signaling	11904S
Rabbit polyclonal anti-CANX	Enzo Life Sciences, Farmingdale, NY	ADI-SPA-865-F
Rabbit polyclonal anti-IFIT1	Abnova, Taipei, Taiwan	H00003434-DO1
Rabbit anti-human-IFIT3 (3 μg/μl, in PBS-glycerin, affinity-purified)	Self-made Pichlmair, Lassnig (1)	N/A
Rabbit anti-NS5B	Volker Lohmann, Heidelberg University Hospital, Germany	N/A
